# Assessment of Vitamin D status and association with inflammation: Biomarkers Reflecting Inflammation and Nutritional Determinants of Anemia (BRINDA) project

**DOI:** 10.1016/j.ajcnut.2022.10.018

**Published:** 2022-12-21

**Authors:** Melissa F. Young, Jiangda Ou, Cam Duong, Hanqi Luo, Yara S. Beyh, Jiawei Meng, Alison D. Gernand, Daniel E. Roth, Parminder S. Suchdev

**Affiliations:** 1 Hubert Department of Global Health, Rollins School of Public Health, Emory University, Atlanta, GA, USA; 2 Department of Nutritional Sciences, The Pennsylvania State University, University Park, PA, USA; 3 Department of Pediatrics and the Centre for Global Child Health, The Hospital for Sick Children, Toronto, ON, Canada; 4 Centers for Disease Control and Prevention, Atlanta, GA, USA

**Keywords:** inflammation, vitamin D, BRINDA, preschool age children, females of reproductive age, micronutrient assessment

## Abstract

**Background:**

It is unclear whether 25(OH)D concentrations in children and female adults may be influenced by inflammation and thus require adjustment when estimating the population prevalence of vitamin D deficiency.

**Objectives:**

We examined correlations between inflammation biomarkers, CRP or alpha-1-acid glycoprotein (AGP), and serum 25(OH)D concentrations among preschool children (PSC; 6–59 mo) and nonpregnant females of reproductive age (FRA; 15–49 y).

**Methods:**

We analyzed cross-sectional data from 6 nationally representative nutrition surveys (Afghanistan, Cambodia, Pakistan, UK, USA, and Vietnam) conducted among PSC (n = 9880) and FRA (n = 14,749) from the Biomarkers Reflecting Inflammation and Nutritional Determinants of Anemia project. Rank correlations between CRP or AGP and 25(OH)D concentrations were examined while taking into account complex survey design effects.

**Results:**

Among both PSC and FRA, correlations between inflammation and vitamin D biomarkers were weak and inconsistent across surveys. For PSC, correlation coefficients between CRP and 25(OH)D concentrations ranged from −0.04 to 0.08, and correlations between AGP and 25(OH)D ranged from 0.01 to 0.05. Correlation coefficients between CRP and 25(OH)D for FRA ranged from −0.11 to 0.14, and correlations between AGP and 25(OH)D concentrations ranged from −0.05 to 0.01.

**Conclusions:**

Based on the weak and inconsistent correlations between CRP or AGP and 25(OH)D, there is no rationale to adjust for these inflammation biomarkers when estimating population prevalence of vitamin D deficiency in PSC or FRA.

## Introduction

Vitamin D deficiency is an important public health problem that can contribute to the loss of bone density leading to osteoporosis and fractures as well as rickets in children [1, 2]. Although population data on vitamin D deficiency prevalence is limited, the available evidence suggests widespread global vitamin D deficiency, particularly in countries where calcium intake is low and vitamin D fortification is not mandatory [3]. Accurate assessment of vitamin D is critical to evaluate the prevalence of vitamin D deficiency, identify at-risk subgroups, and guide public health actions to mitigate the burden of deficiency. Studies worldwide commonly assess vitamin D status using the circulating, intermediate form, 25(OH)D, because of its long half-life, relatively stability, and high plasma concentration. In contrast, the active form of vitamin D, 1,25-dihydroxyvitamin D (1,25(OH)_2_D), has unstable serum concentrations owing to its dependence on calcium concentrations and thus is not considered a good indication of vitamin D status [4]. Disagreement exists about the thresholds to define risk of deficiency; the Institute of Medicine defines deficiency as 25(OH)D below 30 nmol/L [5], whereas the Endocrine Society [6] and European Food Safety Authority [7] recommend a threshold of 50 nmol/L. Debates also exist surrounding laboratory approaches to quantify 25(OH)D concentrations (e.g., radioimmunoassay, chemiluminescent assay or liquid chromatography), making it challenging to choose the cutoff to define deficiency [8–10].

Some studies have also suggested that persistent inflammation and chronic infections may alter the concentration of 25(OH)D [11]. Inflammation is characterized by high circulating concentrations of inflammatory mediators and associated with diverse pathophysiology of various infections and chronic diseases [12]. During inflammation, the activation of toll-like receptors and several cytokines such as IFN-γ can up-regulate vitamin D-binding receptors in macrophages and as a result stimulate a rapid conversion from 25(OH)D to 1,25(OH)_2_D, whereas other cytokines such as IL-4 can induce the catabolism of 25(OH)D to the inactive metabolite 24,25-dihydroxycholecalciferol (24,25(OH)_2_D) [11]. Although there are many different indicators of inflammation, national surveys typically measure acute phase proteins, including CRP and alpha 1-acid glycoprotein (AGP). CRP and AGP are enhanced by several cytokines such as IL-6, IL-1, and TNF (13). The associations of CRP and AGP with biomarkers of iron and vitamin A have been demonstrated in previous studies from the Biomarkers Reflecting Inflammation and Nutritional Determinants of Anemia (BRINDA) project, and as a result adjustment methods were recommended for iron and vitamin A serum concentrations [14].

However, evidence of the association between vitamin D status and biomarkers of inflammation is sparse and conflicting [15–21], and it remains unclear if adjustment is merited. A study in Sri Lanka assessing vitamin D status during infections showed a lower concentrations of 25(OH)D in hospitalized children with dengue symptoms than in healthy children [19], whereas other studies tracking 25(OH)D concentrations during the course of pneumonia infections in children in Nepal [18] or malarial infections in adults in the UK [20] showed no significant changes in 25(OH)D concentrations. Studies assessing the correlations between vitamin D and inflammatory biomarkers have also reported mixed findings. For example, a cohort study of adult patients referred for nutritional assessment and adult patients with critical illnesses in Scotland showed that serum 25(OH)D concentrations were inversely related to CRP concentration [15], and a cross-sectional study of children with obesity in Spain also found an inverse correlation between 25(OH)D and CRP or IL-6 concentrations [16]. In contrast, other studies of patients with inflammatory bowel disease in Germany [17] or periodontal disease in the United States [21] found a stable concentration of 25(OH)D regardless of the presence of elevated CRP. Given the conflicting evidence about the need for inflammation adjustment in vitamin D status assessment, the aim of the present study was to examine whether inflammatory biomarkers (CRP and AGP) are correlated with 25(OH)D among preschool children (PSC) as well as females of reproductive age (FRA) in studies included in the BRINDA project.

## Methods

We used cross-sectional survey data from the BRINDA project (www.BRINDA-nutrition.org). Methods for data acquisition and management and for defining inclusion and exclusion criteria have been previously described [22, 23]. The BRINDA protocol was reviewed by the institutional review boards of the National Institute of Health and was deemed to be non-human-subjects research. In the present study, we included nationally representative surveys in PSC (aged 6–59 mo) and nonpregnant FRA (15–49 y), where the sample size was larger than 100 and data were available for at least one biomarker of inflammation (CRP, AGP, or both) and 25(OH)D (Supplemental Figure 1). After applying these criteria, we obtained 5 data sets that included data from PSC and FRA and an additional data set that included only FRA. Our final data sample included 5 datasets for PCS (*n* = 9880) and 6 for FRA (*n* = 14,749) (Supplemental Table 1).

### Laboratory analysis

Venous blood was obtained from all survey participants. Plasma and serum samples were kept at −20 °C until analysis in all participating surveys. The concentrations of 25(OH)D, CRP, and AGP were measured using several laboratory methods that are summarized for each survey in Supplemental Table 2. Methods for CRP and AGP assessment are mostly consistent across assays owing to the availability of reference materials and external quality assessment programs [26–30]. We defined inflammation using CRP concentration > 5 mg/L or AGP concentration > 1 g/L [24].

### Statistical analysis

We first described the distribution of 25(OH)D, CRP, and AGP concentrations using median and IQR and calculated the prevalence of deficiency by survey. All analyses took into account complex survey design effects, including cluster, strata, and biomarker-specific sampling weights, unless otherwise specified. We calculated weighted rank correlation coefficients because the variables did not necessarily follow normal distribution (Gaussian distribution), and because we did not want to assume that monotonic relationships were linear. This was done by first calculating the ranks of inflammation (CRP or AGP) and 25(OH)D within each survey and then estimating the weighted Pearson’s correlation between the rank variables. The prevalence of 25(OH) D < 30 nmol/L was plotted by unweighted CRP and AGP decile to investigate whether there was a linear relationship between inflammation and 25(OH)D. Results were considered significant at *P* value < 0.05. We categorized BMI values as underweight (BMI < 18.5), normal weight (18.5 ≤ BMI < 25), overweight (25 ≤ BMI < 30), and obesity (BMI ≤ 30) and conducted stratification analyses to examine the potential role of BMI in modifying the associations between inflammation and vitamin D status among FRA. In addition, we built a general linear model and accounted for the complex survey design to test the significance of interaction between BMI category and inflammatory markers on vitamin D, with each survey as a fixed effect. All statistical analyses were performed using SAS 9.4 (SAS Institute) and incorporated complex survey design effects, including cluster, strata, and biomarker-specific sampling weights, as done previously [14, 25].

## Results

### Participant characteristics

Mean age among PSC (*n* = 9880) ranged from 27.4 mo in Pakistan to 37.4 mo in Vietnam, whereas the mean age of FRA (*n =* 14,749) varied between 20.9 y in Afghanistan and 34.4 y in the UK (Table 1). The percentage of individuals included in each survey based on vitamin D biomarker availability was similar to CRP and AGP availability and ranged from 3% in Afghanistan to 93% in USA (Supplemental Table 3). Demographic characteristics were similar among those included and excluded for all surveys, and population groups except for Afghanistan where included PSC and FRA had higher SES than those who were excluded Supplemental Table 4). The prevalence of PSC with elevated CRP, defined as CRP higher than 5 mg/L, was lowest in USA (6.0%) and highest in Vietnam (12.4%). In contrast, the prevalence of elevated CRP among FRA was lowest in Vietnam (7.0%) and highest in USA (25.6%). The prevalence of elevated AGP was higher than that of elevated CRP. It was lowest in Afghanistan (23.7% among PSC or 11.6% among FRA) and highest in Cambodia (36.0% among PSC and 33.6% among FRA). The prevalence of PSC with 25(OH)D lower than 30 nmol/L ranged between 0.8% in USA and 41% Afghanistan, whereas the prevalence among FRA ranged between 5.9% in Cambodia and 84.5% in Afghanistan. Similarly, when using 50 nmol/L cutoff to define vitamin D deficiency, the prevalence of deficiency among PSC ranged between 11.9% in USA and 82.2% in Afghanistan, whereas the prevalence among FRA varied from 30.5% in Cambodia to 92.9% in Afghanistan.

### Relationship between vitamin D and inflammation

**Figure 1.**
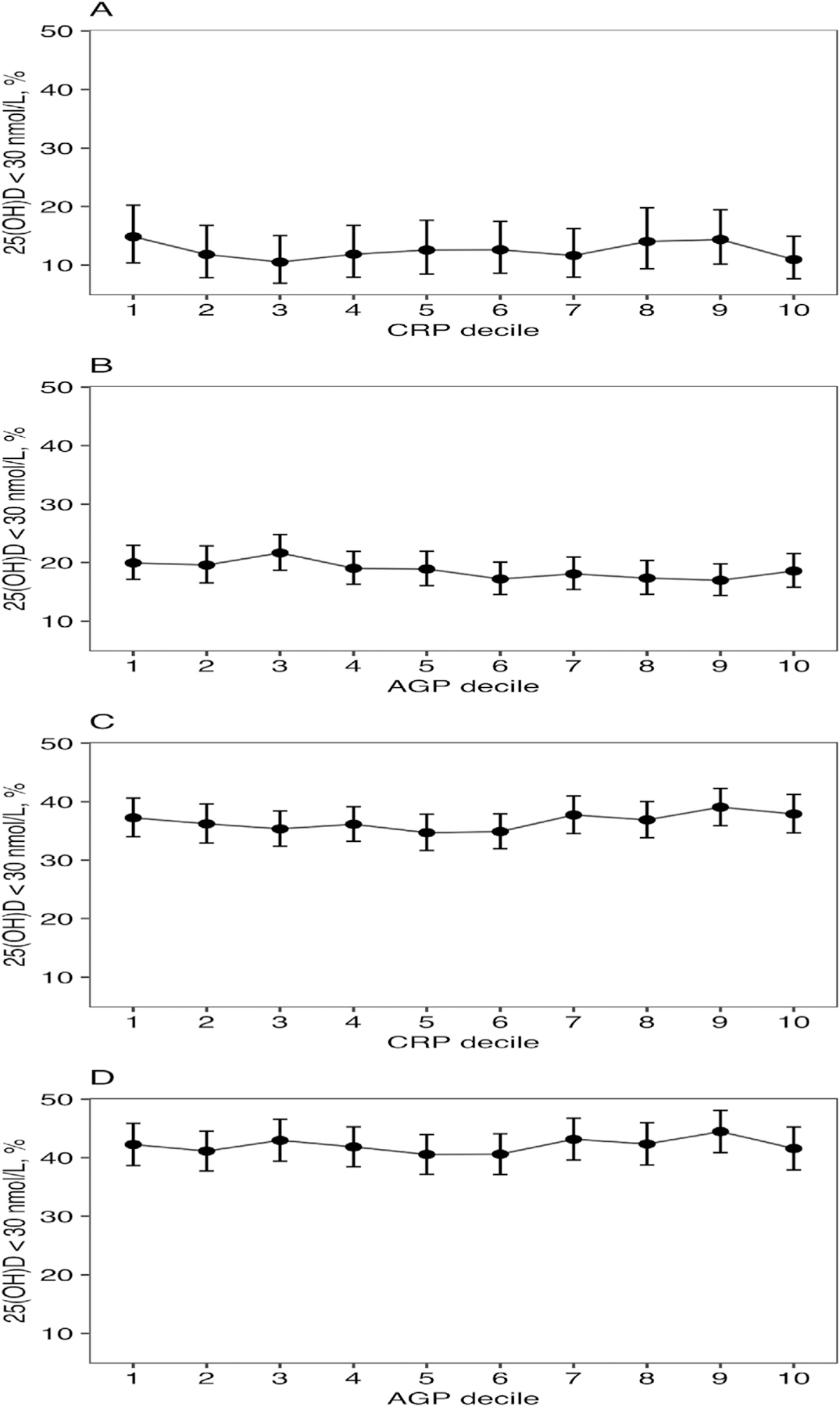
Prevalence of 25(OH)D < 30 nmol/L by (A) CRP deciles and (B) AGP deciles in preschool-age children (*n* = 9880) and prevalence of 25(OH)D < 30 nmol/L by (C) CRP deciles and (D) AGP deciles in females of reproductive age (*n*= 14,749). AGP, alpha-1-acid glycoprotein.

Most surveys had very weak correlations that were not statistically significant between 25(OH)D and inflammatory biomarkers (CRP or AGP) in both PSC and FRA (Table 2). Regarding 25(OH)D and CRP, all surveys of PSC had non-statistically significant associations whereas 3 surveys of FRA in UK, USA, and Vietnam had statistically significant associations (although still small), of which the surveys conducted in the UK and USA had inverse correlations, and the survey conducted in Vietnam had a positive correlation. Correlations between 25(OH)D and AGP concentrations were all non-statistically significant for both PSC and FRA. The assoication between 25(OH)D and CRP or AGP does not consistently vary at different concentrations of 25(OH)D (results not shown). The prevalence of vitamin D deficiency (25(OH)D < 30 nmol/L) varied across the deciles of CRP and AGP; however, there was no evidence of linear trend for PSC or FRA (Figure 1). Similar results were found in country specific analysis compared with the pooled results (results not shown).

In terms of testing for interaction between BMI and inflammatory markers on vitamin D, the interaction term was significant between CRP and BMI category and non-significant between AGP and BMI category (data not shown). In our stratification analysis according to BMI (Table 3), the correlation coefficients were small and non-significant across BMI categories in Afghanistan and Pakistan. In the Vietnam sample, the association was small, positive, and statistically significant in the normal weight group. In contrast, the association was small, negative, and statistically significant in the overweight group in the UK sample as well as in the obesity group in the Cambodia sample. In the US sample, where the overall association was significantly negative, the associations were not significant in the obesity group and significantly positive in the normal weight group.

## Discussion

The result of this large, multicountry analysis showed little evidence of a relationship between biomarkers of inflammation (CRP or AGP) and 25(OH)D concentrations among PSC and FRA. Correlations between CRP and AGP and 25(OH)D concentrations were all close to zero for PSC and FRA samples. Furthermore, the directions of the correlations were inconsistent and most *P* values > 0.05. In addition, in both pooled and country specific samples, there was no clear pattern of vitamin D deficiency across deciles of inflammatory markers. These results suggest that adjusting for these inflammation biomarkers may not be required to estimate vitamin D deficiency in population-based surveys.

The weak and inconsistent correlations between 25(OH)D and CRP or AGP found in our study are in alignment with the conflicting research results to date [17, 18, 26–28]. For example, in our analysis, among PSC there were no clear associations between vitamin D and inflammatory biomarkers, which is consistent with a previous longitudinal analysis of PSC with pneumonia [18]. However, a cross-sectional analysis of children aged between 0 and 8 y in 6 African countries and a randomized trial of adolescents in Iran both found inverse relationships between vitamin D and CRP concentrations [26, 27]. Similarly, we found no consistent associations between vitamin D and inflammatory biomarkers among FRA. Few studies have studied the subgroup of FRA alone; however, the existing studies on adults also showed conflicting results. A recent cross-sectional analysis of older females with periodontal diseases and a randomized trial of female adults with depressive symptoms both found no associations between vitamin D biomarkers and CRP concentrations [21, 29]. In contrast, 2 studies of patients with cardiovascular diseases found an inverse association between vitamin D and high-sensitive CRP biomarkers [28, 30].

These conflicting findings may be due to the variation in age group, illnesses, and severity of inflammation that modify the associations between vitamin D and inflammation. One potential modifying factor is body fat status. Obesity status was correlated with elevated CRP in FRA in previous BRINDA analyses [31]. In our analysis of the UK survey, we found that the negative correlations between 25(OH)D and CRP in the overall FRA sample may have been driven by the negative associations found in the overweight group. In the NHANES, although the overall correlations between 25(OH)D and CRP were weak and negative, there were non-significant negative correlations in the obesity group but positive significant correlations in the normal BMI group. Previous analyses of the NHANES surveys in US have reported negative associations [32, 33]. A cross-sectional study of pregnant females in China indicated a modifying relationship between lipid profile, vitamin D, and CRP suggesting that high 25(OH)D concentrations prevent the rise of high-sensitive CRP induced by high concentrations of lipid biomarkers [34]. A study of US adult patients with cardiovascular diseases also found positive association between BMI and high-sensitive CRP [30], and another study of Tanzanian adult patients with tuberculosis reported a positive association between BMI and vitamin D [35]. Several mechanisms can account for the relationship between body fat, vitamin D, and inflammation such as increased uptake of vitamin D by adipose tissue, reduced sun exposure that is indispensable for cutaneous vitamin D synthesis, and negative feedback loop on hepatic synthesis of vitamin D [36]; however, more studies with more sensitive measurements of body fat are needed to investigate the complex relationship between body fat, vitamin D, and inflammation.

The justification to adjust estimates of the prevalence of vitamin D deficiency for inflammation requires a strong biologic basis; however, our understanding remains limited about the bidirectional relationship between vitamin D and inflammation. Many have postulated that vitamin D deficiency is the consequence of inflammation, possibly owing to the rapid renal conversion between prohormone 25(OH)D to 1,25(OH)_2_D which is up-regulated during infection or inflammation [17, 37]. Evidence supporting this hypothesis remained mixed as several studies observed that the concentration of 25(OH)D dropped after inflammation [28], whereas other studies found that the concentration of 25(OH)D was relatively stable during and after the course of infection and inflammation [18, 20, 38]. In contrast, others have argued that vitamin D deficiency can contribute to inflammation through promoting the synthesis of antimicrobial peptides, up-regulating anti-inflammatory cytokines, activating adaptive immune response, and modulating cytosol calcium concentrations [37]. Furthermore, excess 25(OH)D concentrations could also lead to proinflammatory effects and increase the risk of chronic diseases [39, 40]. The biological mechanisms linking vitamin D and inflammatory biomarkers need to be better understood before justifying the adjustment of vitamin D biomarkers for inflammation.

At least 99% of the 25(OH)D in serum is bound to vitamin D-binding protein (VDBP) or albumin, both of which are negative acute phase reactants [41, 42]. Therefore, declines in serum VDBP and albumin concentrations may contribute to a decline in the total 25(OH)D with inflammation [41]; however, VDBP data were not available in the datasets included in this study to examine this mechanism. Measurement of unbound (“free”) 25(OH)D or bioavailable 25(OH)D has been proposed as an alternative biomarker of vitamin D status that may be less affected by variations in VDBP or albumin concentrations [43, 44]. However, total 25(OH)D (including bound and unbound fractions) is a standard biomarker of vitamin D status, whereas the measurement of the much reduced, free or bioavailable 25(OH)D concentration is challenging, not standardized and not widely implemented in epidemiological studies or population surveys [43].

The key strength of this study is the large and diverse populationbased data that included several countries in Asia, one from Europe and one from America regions, in contrast to most existing studies that are clinical-based. Our large sample, including close to 10,000 PSC and 15,000 FRA, lend a strong statistical power to detect the assoication between inflammation and vitamin D and enable a more comprehensive understanding of the common patterns or divergence in the relationships between different contexts. However, the study had several limitations. First, our findings are limited to FRA and PSC population-based surveys and cannot be generalizable to other populations groups (school-aged children or pregnant females), or clinical settings, which merits further examination. Second, we lack data on other biomarkers of inflammation such as ILs and tumor necrosis factor, which correlate with vitamin D status in studies of healthy adolescents [27] or patients with heart failure [45]. Future studies should look at a range of inflammatory biomarkers to better understand its reciprocal relationship with vitamin D. Third, our analysis was constrained by the variability of the conditions and methods that were used to obtain blood samples and analyze vitamin D, CRP, and AGP concentrations. In particular, we lacked information about CRP in the data sets, for which the limit of detection (LOD)/limit of quantification (LOQ) can vary widely across assays and for which a high proportion of undetectable or unquantifiable observations may be observed. Unfortunately, information on the LODs and LOQs was not available for all surveys, and future nutrition surveys should include information on LOD/LOQ in survey reports and publications to allow for standardized approach across datasets. Furthermore, the low proportion of individuals included in the analysis based on biomarker availability in some countries could have generated selection bias. Lastly, random measurement error on single measurements of biomarkers may attenuate correlations coefficients. Future research would benefit from longitudinal designs with repeat measurements of biomarkers.

In conclusion, our multicountry analysis of nationally representative surveys found no evidence to justify the adjustment of 25(OH)D concentrations by CRP and AGP among PSC and FRA in population-based surveys. There is a need for better understanding of the reciprocal relationships between vitamin D and inflammation and standardization of laboratory methods across surveys to enable measurement of reliable biomarkers.

We are grateful for the contributions of datasets and guidance from the BRINDA working group and steering committee members; the BRINDA steering committee consists of (in alphabetical order): Erick Boy, Rafael Flores, Janet King, Sorrel Namaste, Lynnette Neufeld, Daniel Raiten, Parminder Suchdev, James Wirth, and Melissa F Young. We acknowledge the contributions of the BRINDA working group and country representatives (https://brinda-nutrition.org/). We also appreciate the contributions of Jiaxi Geng, Chelsea Cole, and Emily Nieckula. The authors’ responsibilities were as follows – MFY, JO, ADG, DR, and PS: designed research; JO, HL, JM, and MFY: analyzed data; MFY, MCD, and YSB: wrote paper; MFY: had primary responsibility for final content (MFY); and all authors: read and approved the final manuscript. The authors report no conflicts of interest.

## Supplementary Material

appendix

## Figures and Tables

**Table 1:** Age, inflammation and vitamin D status of preschool children and nonpregnant females of reproductive age, BRINDA project^1^

			CRP		AGP		Vitamin D		
Survey, year	*N*	Age^2^ Mean (95% CI)	CRP Median (IQR), mg/L	CRP % (95% CI) > 5 mg/L	AGP Median (IQR), g/L	AGP % (95% CI) > 1 g/L	25(OH)D Median (IQR), nmol/L	25(OH)D % (95% CI) < 30 nmol/L	25(OH)D % (95% CI) < 50 nmol/L
**PSC**									
Afghanistan, 2013	662	29.1 (27.7, 30.4)	0.15 (0.0, 1.1)	10.1 (6.9, 13.3)	0.8 (0.6, 1.0)	23.7 (19.5, 28.0)	34.0 (23.3, 45.4)	41.4 (35.0, 47.7)	82.2 (76.9, 87.5)
Cambodia, 2014	646	36.4 (35.3, 37.5)	0.4 (0.2, 1.3)	10.1 (7.3, 12.9)	0.7 (0.5, 1.4)	36.0 (29.4, 42.6)	89.1 (61.5, 116.7)	4.0 (1.4, 6.7)	14.4 (8.1, 20.7)
Pakistan, 2011	6943	27.4 (26.9, 27.8)			0.9 (0.7, 1.1)	35.3 (33.8, 36.8)	58.7 (36.6, 85.4)	18.0 (16.5, 19.5)	40.1 (38.0, 42.2)
USA, 2006	1314	36.7 (35.6, 37.8)	0.2 (0.1, 0.6)	6.0 (4.5, 7.5)	—	—	69.0 (58.1, 80.7)	0.8 (0.4, 1.2)	11.9 (9.4, 14.3)
Vietnam, 2010	315	37.4 (36.0, 38.8)	0.5 (0.2, 1.3)	12.4 (9.0, 15.7)	—	—	46.7 (32.7, 61.3)	21.0 (16.0, 25.9)	56.5 (51.0, 62.0)
**FRA**									
Afghanistan, 2013	1044	20.9 (30.3, 31.5)	1.0 (0.2, 2.7)	12.9 (10.5, 15.3)	0.7 (0.6, 0.9)	11.6 (8.8, 14.5)	16.4 (11.6, 24.0)	84.5 (81.6, 87.4)	92.9 (90.7, 95.1)
Cambodia, 2014	699	30.2 (29.5, 30.9)	0.7 (0.3, 1.8)	9.5 (7.2, 11.9)	0.7 (0.5, 1.3)	33.6 (25.0, 42.3)	63.5 (46.1, 85.7)	5.9 (2.8, 8.9)	30.5 (23.8, 37.3)
Pakistan, 2011	8387	30.9 (30.8, 31.1)	0.9 (0.3, 2.3)	11.8 (10.8, 12.8)	0.8 (0.7, 1.0)	23.6 (22.3, 24.8)	37.3 (21.2, 59.1)	38.8 (36.8, 40.8)	66.7 (64.8, 68.6)
UK, 2014	894	34.4 (33.5, 35.3)	1.9 (1.3, 3.2)	15.5 (12.2, 18.8)	—	—	43.0 (26.4, 62.1)	30.2 (25.7, 34.8)	59.6 (54.3, 64.9)
USA, 2006	3197	33.5 (33.0, 34.0)	1.9 (0.5, 5.1)	25.6 (23.5, 27.8)	—	—	55.8 (40.0, 72.8)	13.9 (10.9, 16.8)	41.0 (36.3, 45.7)
Vietnam, 2010	528	32.8 (31.9, 33.7)	0.7 (0.3, 1.5)	7.0 (4.9, 9.1)	—	—	46.9 (34.5, 57.4)	17.6 (13.9, 21.3)	57.8 (52.8, 62.7)

1 AGP, alpha-1-acid glycoprotein; BRINDA, Biomarkers Reflecting Inflammation and Nutritional Determinants of Anemia; and — not measured.

2 Age values are shown in months for preschool children and years for females of reproductive age.

**Table 2:** Weighted rank correlations between 25(OH)D and CRP and AGP in preschool children and nonpregnant females of reproductive age, the BRINDA project^1,2^

Survey	*n*	25(OH)D and CRP correlation		n	25(OH)D and AGP correlation	
		*r*	*p*		*r*	*p*
**PSC**						
Afghanistan, 2013	662	−0.04	0.50	662	0.04	0.61
Cambodia, 2014	646	0.04	0.32	646	0.05	0.29
Pakistan, 2011				6943	0.01	0.63
USA, 2006	1314	0.01	0.78	—	—	—
Vietnam, 2010	315	0.08	0.08	—	—	—
**FRA**						
Afghanistan, 2013	1044	0.01	0.92	1044	0.01	0.81
Cambodia, 2014	699	0.05	0.40	699	−0.05	0.47
Pakistan, 2011	6305	0.001	0.92	8045	−0.01	0.56
UK, 2014	894	−0.11	0.02	—	—	—
USA, 2006	3197	−0.10	0.001	—	—	—
Vietnam, 2010	528	0.14	0.002	—	—	—

1 AGP, alpha-1-acid glycoprotein; BRINDA, Biomarkers Reflecting Inflammation and Nutritional Determinants of Anemia; and — not measured.

2 *P* value was calculated from T test in regression model that took into account complex sampling (cluster, strata, and sampling weight).

**Table 3:** Weighted rank correlation between 25(OH)D and CRP/AGP by country and BMI category nonpregnant females of reproductive age, the BRINDA project^1^

BMI Category		Afghanistan, 2013		UK, 2014		Cambodia, 2014		Pakistan, 2011		USA, 2006		Vietnam, 2010	
		25(OH)D and CRP correlation	25(OH)D and AGP correlation	25(OH)D and CRP correlation	25(OH)D and AGP correlation	25(OH)D and CRP correlation	25(OH)D and AGP correlation	25(OH)D and CRP correlation	25(OH)D and AGP correlation	25(OH)D and CRP correlation	25(OH)D and AGP correlation	25(OH)D and CRP correlation	25(OH)D and AGP correlation
**Underweight**	n	43	43	26	—	94	94	984	1297	124	—	107	—
	*r*	−0.23	−0.02	0.09	—	−0.02	−0.02	0.02	−0.001	0.2	—	0.17	—
	p	0.21	0.94	0.77	—	0.83	0.88	0.51	0.98	0.08	—	0.08	—
**Normal**	n	372	372	424	—	468	468	3299	4213	1323	—	375	—
	*r*	−0.04	−0.05	−0.02	—	0.06	−0.05	0.02	0.01	0.15	—	0.11	—
	p	0.61	0.49	0.82	—	0.4	0.51	0.3	0.7	0.001	—	0.03	—
**Overweight**	n	140	140	236	—	107	107	1332	1679	746	—	42	—
	*r*	−0.01	−0.02	−0.24	—	0.12	0.01	−0.01	−0.002	0.09	—	0.001	—
	p	0.88	0.87	0.006	—	0.33	0.96	0.6	0.95	0.06	—	0.99	—
**Obesity**	n	57	57	196	—	30	30	648	806	969	—	4	—
	*r*	−0.11	−0.03	0.01	—	−0.41	−0.33	−0.07	−0.02	−0.05	—	0	—
	p	0.42	0.83	0.86	—	0.006	0.03	0.14	0.69	0.26	—	1	—

1 AGP, alpha-1-acid glycoprotein; — not measured.

## Data Availability

Data described in the manuscript, code book, and analytic code will be made available upon request, pending approval from the BRINDA steering committee and country representatives.

## Abbreviations used:

AGP: alpha-1-acid glycoprotein
BRINDA: Biomarkers Reflecting Inflammation and Nutritional Determinants of Anemia
PSC: preschool children
FRA: females of reproductive age
