# Supplementary material for: Assessment of Vitamin D status and association with inflammation: Biomarkers Reflecting Inflammation and Nutritional Determinants of Anemia (BRINDA) project
Source: Am J Clin Nutr. Author manuscript; Available in PMC 2023 May 22. (PMC10202120; doi:10.1016/j.ajcnut.2022.10.018)
Supplement: appendix [file NIHMS1898154-supplement-appendix.docx]

Online Supporting Material (OSM)

**Assessment of Vitamin D status and association with inflammation: Biomarkers Reflecting Inflammation and Nutritional Determinants of Anemia (BRINDA) project.** Melissa F. Young

**BRINDA Phase 1 & 2**

24 PSC datasets (N=85,458);

19 FRA datasets (N= 107,393)

**Inclusion Criteria**

At least one inflammation biomarker *AND*

25(OH)D *AND* Dataset *n* > 100

**Supplemental Figure 1: BRINDA Project Available Data for Vitamin D and Inflammation**

**Data Included in Analysis**

5 PSC datasets (N = 9,880);

6 FRA datasets (N = 14,749)

Online Supporting Material (OSM)

**Supplemental Table 1: data source of the survey used in the analysis**

| **Survey, year** | **Population group** | **Survey name** | **Citation** |
| --- | --- | --- | --- |
| Afghanistan, 2013 | FRA, PSC | National Nutrition Survey Afghanistan | Ministry of Public Health (Afghanistan); United Nations Children’s Fund (UNICEF); Aga Khan University; Central Statistics Organization (Afghanistan); Silk Route Training and Research Organization (SRTRO). National Nutrition Survey Afghanistan; 2013. |
| Cambodia, 2014 | FRA, PSC | Cambodia Demographic Health Survey | National Institute of Statistics; Directorate General for Health; ICF International. Cambodia Demographic and Health Survey 2014; 2015. |
| Pakistan, 2011 | FRA, PSC | Pakistan National Nutrition Survey 2011 | Bhutta, Z. A.; Soofi, S. B.; Zaidi, S. S.; Habib, A. Pakistan National Nutrition Survey 2011; 2011. |
| United Kingdom, 2014 | FRA | National Diet and Nutrition Survey Results | Public Health England; Food Standards Agency. National Diet and Nutrition Survey Results from Years 5 and 6 (Combined) of the Rolling Programme (2012/2013 - 2013/2014); 2016. |
| USA, 2006 | FRA, PSC | National Health and Nutrition Examination Survey 1999-2018 | CDC. Second National Report on Biochemical Indicators of Diet and Nutrition in the US Population; 2012. |
| Vietnam, 2010 | FRA, PSC | 2010 Micronutrient Status Survey | Laillou, A.; Van Pham, T.; Tran, N. T.; Le, H. T.; Wieringa, F.; Rohner, F.; Fortin, S.; Bach Le, M.; Tran, D. T.; Moench-Pfanner, R.; Berger, J. Micronutrient Deficits Are Still Public Health Issues among Women and Young Children in Vietnam. PLoS ONE 2012, 7 (4). |

Online Supporting Material (OSM)

**Supplemental Table 2. Biomarker availability and lab methods by survey^1^**

| **Survey, year** | **Survey name** | **Vitamin D**  **25(OH)D** | **CRP** | **AGP** |
| --- | --- | --- | --- | --- |
| Afghanistan, 2013 | National Nutrition Survey Afghanistan | CLIA | Immunoassay | TIA |
| Cambodia, 2014 | Cambodia Demographic Health Survey | ECLIA | Sandwich ELISA | Sandwich ELISA |
| Pakistan, 2011 | Pakistan National Nutrition Survey 2011 | CLIA | NA | Turbidimetry |
| United Kingdom, 2014 | National Diet and Nutrition Survey | CLIA^2^ | PETIA | NA |
| USA, 2006 | National Health and Nutrition Examination Survey | RIA^2^ | Nephelometry | NA |
| Vietnam, 2010 | 2010 Micronutrient Status Survey | HPLC^2^ | ELISA | NA |

^1^ **Abbreviations:** Alpha-1-acid glycoprotein (AGP), chemiluminescent immunoassay (CLIA), C-reactive protein (CRP), electrochemiluminescence immunoassay (ECLIA), enzyme-linked immunosorbent assay (ELISA), high performance liquid chromatography (HPLC), red blood cell (RBC), particle enhanced turbidimetric immunoassay (PETIA)**,** radioimmunoassay (RIA), turbidimetric immunoassay (TIA).

^2^ Studies participated in external quality assurance.

Online Supporting Material (OSM)

**Supplementary Table 3: Sample sizes for key biomarkers in preschool-age children and non-pregnant females of reproductive age, BRINDA project**

|  |  | AGP | | | CRP | | Vitamin D | | Inclusion data | |
| --- | --- | --- | --- | --- | --- | --- | --- | --- | --- | --- |
| Survey, year | Total n | | n | % | n | % | n | % | n | % |
| **Preschool-age children** | | | | | | | | | | |
| Afghanistan, 2013 | 19896 | | 665 | 3 | 665 | 3 | 662 | 3 | 662 | 3 |
| Cambodia, 2014 | 874 | | 665 | 76 | 665 | 76 | 647 | 74 | 646 | 74 |
| Pakistan, 2011 | 10689 | | 7557 | 71 | 0 | 0 | 7300 | 68 | 6943 | 65 |
| USA, 2006 | 2665 | | 0 | 0 | 1315 | 49 | 1465 | 55 | 1314 | 49 |
| Vietnam, 2010 | 395 | | 0 | 0 | 378 | 96 | 317 | 80 | 315 | 80 |
| **Non-pregnant females of reproductive age** | | | | | | | | | | |
| Afghanistan, 2013 | 23875 | | 1050 | 4 | 1050 | 4 | 1044 | 4 | 1044 | 4 |
| Cambodia, 2014 | 724 | | 705 | 97 | 705 | 97 | 703 | 97 | 699 | 97 |
| Pakistan, 2011 | 22278 | | 8261 | 37 | 7897 | 35 | 8593 | 39 | 8387 | 38 |
| United Kingdom, 2014 | 2050 | | 0 | 0 | 942 | 46 | 916 | 45 | 894 | 44 |
| USA, 2006 | 3456 | | 0 | 0 | 3197 | 93 | 3199 | 93 | 3197 | 93 |
| Vietnam, 2010 | 1492 | | 0 | 0 | 1483 | 99 | 531 | 36 | 528 | 35 |

Online Supporting Material (OSM)

**Supplementary Table 4: Demographic and socioeconomic status in all data and included data in preschool-age children and non-pregnant females of reproductive age, BRINDA project**

| **Survey, year** | **variable** | **Preschool-age children** | | **Non-pregnant women of reproductive age** | |
| --- | --- | --- | --- | --- | --- |
|  |  | **All data** | **Inclusion** | **All data** | **Inclusion** |
| Afghanistan, 2013 | Age | 30 (15) | 28 (14) | 28 (10) | 31 (8) |
|  | Male | 51.3% (10216) | 52.6% (348) | -- | -- |
|  | Low SES | 40.7% (8082) | 13.2% (87) | 36.4% (8661) | 14.9% (155) |
|  | Middle SES | 40.9% (8129) | 38.8% (255) | 40.3% (9578) | 36.9% (383) |
|  | High SES | 18.4% (3654) | 47.9% (315) | 23.3% (5545) | 48.1% (499) |
| Cambodia, 2014 | Age^1^ | 33(16) | 36(15) | 30(7) | 30(6) |
|  | Male | 53.4% (467) | 55.4% (358) | -- | -- |
|  | Rural | 78.1% (683) | 78.2% (505) | 79.4% (575) | 79.7% (557) |
|  | Low SES | 41.5% (363) | 44.1% (285) | 42.7% (309) | 42.8% (299) |
|  | Middle SES | 39.1% (342) | 39.3% (254) | 36.6% (265) | 36.8% (257) |
|  | High SES | 19.3% (169) | 16.6% (107) | 20.7% (150) | 20.5% (143) |
| Pakistan, 2011 | Age | 27(15) | 27(15) | 32(7) | 31(6) |
|  | Male | 52.2% (5583) | 51.8% (3595) | -- | -- |
|  | Rural | 60.2% (6435) | 60.6% (4206) | 59.2% (13188) | 60.5% (5070) |
|  | Low SES | 41.0% (4379) | 40.7% (2823) | 40.4% (8992) | 39.9% (3349) |
|  | Middle SES | 41.0% (4383) | 41.6% (2888) | 40.4% (9005) | 41.9% (3516) |
|  | High SES | 18.0% (1927) | 17.7% (1232) | 19.2% (4281) | 18.1% (1522) |
| United Kingdom, 2014 | Age | NA | NA | 30(11) | 33(11) |
|  | Low SES | NA | NA | 37.4% (672) | 37.2% (297) |
|  | Middle SES | NA | NA | 29.2% (525) | 28.8% (230) |
|  | High SES | NA | NA | 33.4% (600) | 34.0% (272) |
| USA, 2006 | Age | 28(16) | -- | -- | 29(11) |
|  | Male | 49.9% (1329) | 49.8% (654) |  |  |
|  | Low SES | 55.2% (1396) | 56.2% (709) | 39.0% (1280) | 39.5% (1203) |
|  | Middle SES | 32.6% (824) | 32.4% (409) | 39.0% (1279) | 38.5% (1172) |
|  | High SES | 12.3% (310) | 11.3% (143) | 22.0% (721) | 22.0% (670) |
| Vietnam, 2010 | Age | 37(13) | 37(13) | 32(10) | 33(10) |
|  | Male | 53.0% (209) | 53.5% (168) | -- | -- |
|  | Rural | 53.4% (211) | 55.2% (174) | 51.2% (764) | 50.4% (266) |

^1^Age in months in preschool-age children and years in non-pregnant females of reproductive age
